# Association of Alpha Tocopherol and Ag Sulfadiazine Chitosan Oleate Nanocarriers in Bioactive Dressings Supporting Platelet Lysate Application to Skin Wounds

**DOI:** 10.3390/md16020056

**Published:** 2018-02-09

**Authors:** Maria Cristina Bonferoni, Giuseppina Sandri, Silvia Rossi, Eleonora Dellera, Alessandro Invernizzi, Cinzia Boselli, Antonia Icaro Cornaglia, Claudia Del Fante, Cesare Perotti, Barbara Vigani, Federica Riva, Carla Caramella, Franca Ferrari

**Affiliations:** 1 Department of Drug Sciences, University of Pavia, 27100 Pavia, Italy; giuseppina.sandri@unipv.it (G.S.); silvia.rossi@unipv.it (S.R.); Eleonora.Dellera@bouty.it (E.D.); alessandro.invernizzi01@universitadipavia.it (A.I.); cinzia.boselli@unipv.it (C.B.); barbara.vigani@unipv.it (B.V.); carla.caramella@unipv.it (C.C.); franca.ferrari@unipv.it (F.F.); 2 Department of Public Health Experimental and Forensic Medicine, University of Pavia, 27100 Pavia, Italy; antonia.icaro@unipv.it (A.I.C.); federica.riva01@unipv.it (F.R.); 3 Immunohaematology and Transfusion Service and Cell Therapy Unit of Fondazione IRCCS, S. Matteo, 27100 Pavia, Italy; c.delfante@smatteo.pv.it (C.D.F.); c.perotti@smatteo.pv.it (C.P.)

**Keywords:** chitosan oleate, wound healing, platelet lysate, platelet gel, α tocopherol, Ag sulfadiazine, nanocarriers

## Abstract

Chitosan oleate was previously proposed to encapsulate in nanocarriers some poorly soluble molecules aimed to wound therapy, such as the anti-infective silver sulfadiazine, and the antioxidant α tocopherol. Because nanocarriers need a suitable formulation to be administered to wounds, in the present paper, these previously developed nanocarriers were loaded into freeze dried dressings based on chitosan glutamate. These were proposed as bioactive dressings aimed to support the application to wounds of platelet lysate, a hemoderivative rich in growth factors. The dressings were characterized for hydration capacity, morphological aspect, and rheological and mechanical behavior. Although chitosan oleate nanocarriers clearly decreased the mechanical properties of dressings, these remained compatible with handling and application to wounds. Preliminary studies in vitro on fibroblast cell cultures demonstrated good compatibility of platelet lysate with nanocarriers and bioactive dressings. An in vivo study on a murine wound model showed an accelerating wound healing effect for the bioactive dressing and its suitability as support of the platelet lysate application to wounds.

## 1. Introduction

Chitosan is well known in the literature for its numerous biological effects, such as mucoadhesion and permeation enhancement [1,2], antimicrobial activity [3,4], and hemostatic and analgesic action [5]. It is also endowed with wound healing promotion effect, due to its ability to enhance growth factor and cytokine expression, and to promote the stability of growth factors [6,7,8,9,10]. 

Skin wound healing is the result of a series of molecular and cellular processes that involve, in a first phase, secretion of pro-inflammatory cytokines and invasion of the wound by neutrophils and monocytes. This is followed by migration of keratinocytes, fibroblasts and endothelial cells that initiate the tissue remodeling. Large amounts of reactive oxygen species (ROS) are secreted in the first phase by immune cells to support their action towards invading microorganisms. This ROS production has positive role as they mediate pro-inflammation cytokine secretion and angiogenesis [11]. If ROS remain at high concentration for prolonged periods, however, they impair healing and lead to tissue damages due to oxidation of membrane lipids and cell macromolecules. This happens in the case of chronic wounds and diabetic ulcers and suggests the utility of antioxidant agents in these pathologies [12]. 

Among the more recent therapeutic strategies for chronic wounds, there is the topical employment of platelet derivatives, whose therapeutic role in tissue regeneration, due to the release of growth factors (GFs) and cytokines, is widely recognized [13]. Some studies highlight moreover the relationship between platelet GFs and the control of oxidative stress in wound healing. Coskun et al. evidenced that EGF administration in oral mucosa wounds corresponded to a faster decrease of the levels of NO and decrease in lipid peroxidation in comparison with untreated controls. A scavenging activity of epidermal growth factor versus toxic oxidation products was suggested [14]. Similar results were obtained also in skin wounds in a rat excision model, where a correlation was found between the EGF therapy and thiobarbituric acid reactive species (TBARS) decrease [15]. More recently, it has been demonstrated that the administration of the platelet derived growth factor PDGF-BB, an important mediator in the healing of diabetic foot, modifies TBARS and non-enzymatic antioxidant levels in diabetic wound healing [16]. 

In the literature, different platelet derivatives are described and used in wounds, such as platelet rich plasma (PRP) [17], platelet gel (PG) [18,19], and platelet lysate (PL) [20]. Platelet lysate can be easily obtained from plasma apheresis by freezing–thawing cycles, and it has been previously proposed for the treatment of buccal mucosities [21] and corneal ulcer healing [22,23]. To favor the PL employment in skin wound healing, some associations with dressings have been previously proposed [24,25]. 

The aim of the present work was the development of a bioactive dressing containing silver sulfadiazine (AgSD) as anti-infective drug and alpha tocopherol (αTph) as antioxidant agent, and intended to be loaded with autologous PL in the treatment of chronic skin wounds. 

As both AgSD and αTph are poorly soluble, nanocarriers such as polymeric micelles (AgSD-Mic) and nanoemulsions (αTphNE) based on an amphiphilic salt of chitosan with oleic acid, have been previously developed and characterized to improve their dispersion in aqueous environment. In particular, AgSD-Mic of about 150–200 nm dimensions increased AgSD concentration 100 times with respect to saturation solubility [26]. αTphNE allowed obtaining αTph concentration in aqueous dispersion up to 1 mg/mL in 200–250 nm nanoemulsion [27]. AgSD is a broad spectrum anti-infective drug largely used in wound treatment, with uncommon serious side effects. Some concerns of argyria after topical administration arise only at high concentrations, but not at the usual therapeutic dosages, that however can induce some long lasting accumulation of silver in deep tissues [28]. Some authors put in evidence that AgSD can retard wound healing, due to cytotoxic effect and delay in growth factor production, so that advantages can be obtained from the co-administration of AgSD and epidermal growth factor [29]. Compatibility of AgSD with fibroblasts and with the GFs of platelet lysate can be critical, but previous papers demonstrated that the AgSD encapsulated either in SLN or in chitosan oleate nanoparticles maintained its antimicrobial activity and improved its compatibility with PL GFs [26,30]. The relevance of αTph as antioxidant agent in the wound healing promotion has been suggested by the importance of the ROS regulation in chronic wounds as described in the literature [31,32,33]. In a recent study, a stimulating effect of αTphNE on fibroblast and keratinocyte proliferation was observed [27]. Previous studies demonstrated moreover that chitosan oleate itself has some biological activities useful to application in wounds, such as keratinocyte stimulation and anti-infective activity [27,34]. 

AgSD-Mic and αTphNE nanocarriers were loaded in freeze dried dressings able to support PL application to wounds. The biocompatibility of αTphNE with PL was assessed in fibroblast cell cultures. αTphNE and AgSD-Mic were prepared to improve colloidal concentration and dispersion of the active molecules in the aqueous environment during the preparation of the dressings and at the wound site. They were therefore added to chitosan glutamate solution and freeze dried to obtain bioactive dressings (BD). The dressings were characterized for mechanical resistance, morphological aspect, hydration profiles and rheological behavior after hydration, properties important for their ability to protect the wound and to absorb exudates. The dressing was hydrated at the wound site with PL and its ability to support it in wound healing was assessed in vivo in a burn rat model. 

## 2. Results and Discussion

### 2.1. PL and PG Activity on Burn Rat Model

**Figure 1:**
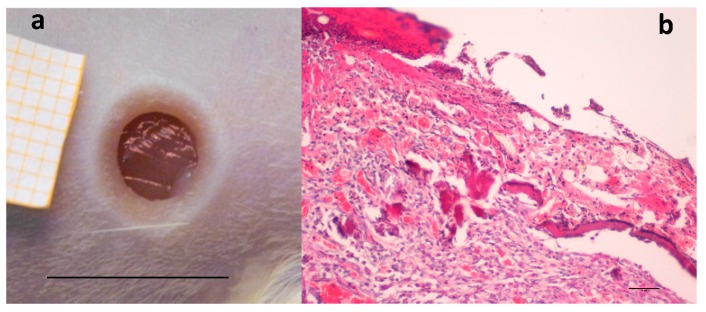
(**a**) Skin full-thickness excisional wound lesion obtained by contact with the brass rod (Ø 4 mm 105 °C) and outlined using a punch biopsy tool 24-h later, bar scale: 10 mm; and (**b**) hematoxylin–eosin staining of the lesion at the same time, scale bar: 200 µm.

A preliminary study was performed to confirm, in the rat wound model used [35], the efficacy of two hemoderivatives, platelet lysate (PL) and platelet gel (PG), well known for their positive effect in accelerating wound healing [18,36]. Figure 1a shows the wound obtained by removing the eschar 24 h after the burn due to application of the brass rod. The lesion is characterized by strong inflammation, consequent to burn injury, as it is possible to see in Figure 1b, and healing is going to occur by second intention. 

**Figure 2:**
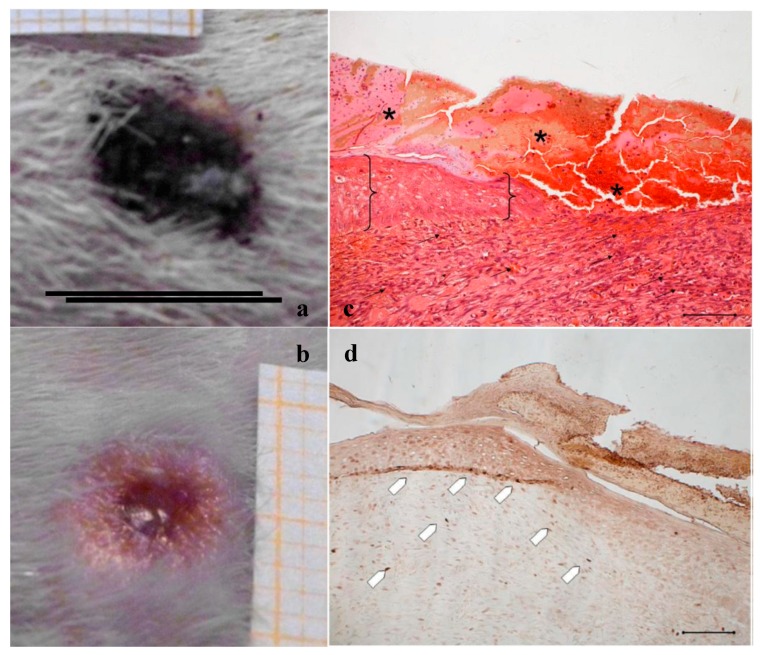
Treatment with NaCl 0.9% *w*/*v* as control. Representative images of wound areas after: (**a**) 14 days; and (**b**) 18 days. Scale bar: 10 mm. Slice sections of the optical microscope after 18 days: (**c**) Hematoxylin–eosin; and (**d**) BrdU staining. Scale bar: 200 μm.

**Figure 3:**
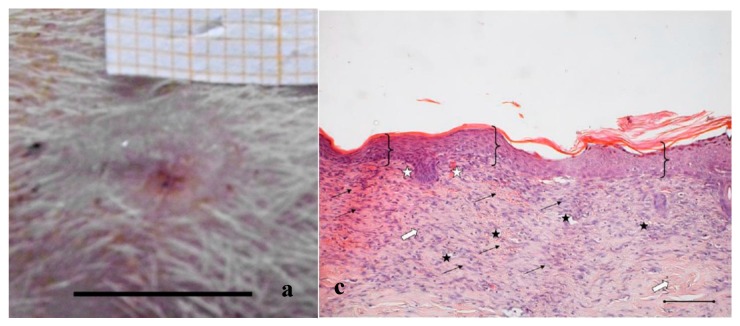

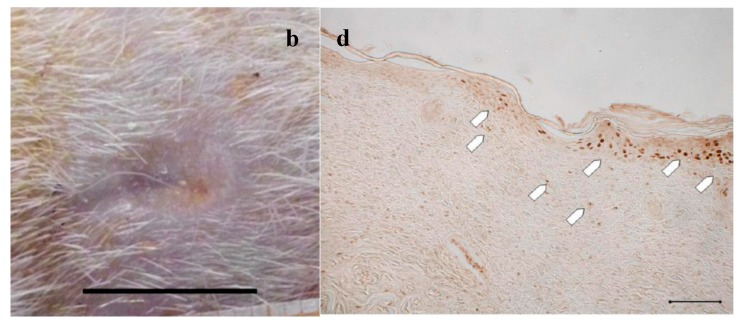
Treatment with PL. Representative images of wound areas after: (**a**) 14 days; and (**b**) 18 days. Scale bar: 10 mm. Slice sections of the optical microscope after 18 days: (**c**) Hematoxylin–eosin; and (**d**) BrdU staining. Scale bar: 200 μm.

**Figure 4:**
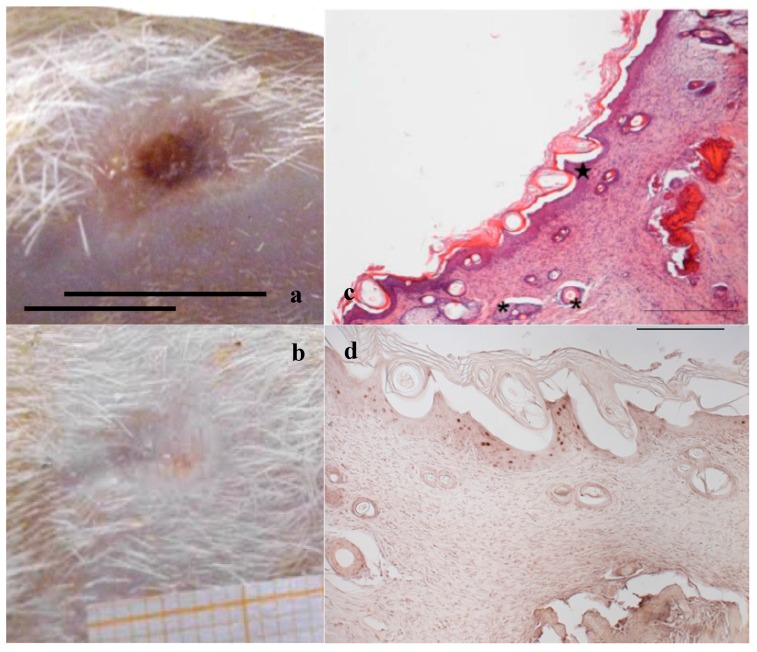
Treatment with PG. Representative images of wound areas: (**a**) after 14 days; and (**b**) 18 days. Scale bar: 10 mm. Slice sections of the optical microscope after 18 days: (**c**) Hematoxylin–eosin; and (**d**) BrdU staining. Scale bar: 200 μm.

In Figure 2, Figure 3 and Figure 4, the macroscopic images of the wounds at 14 and 18 days and the histological evaluation at 18 days are given for the control, and for the PL and the PG treatments. 

Figure 2a,b shows, for the treatment with saline solution (control), the presence at 14 days of an unresolved wound (Figure 2a) that partially persists after 18 days (Figure 2b). The histological evaluation (Figure 2c) of sections stained with hematoxylin–eosin (H&E) indicates that at 18 days in the injured area the epidermis has not yet formed; a large part of epidermis is still replaced by an eosinophilic necrotic tissue (asterisk); in the peripheral regions of the lesion, epidermis (bracket) is visible. In the dermal connective, inflammatory infiltrate (black arrows) is detectable. No skin appendages are visible.

In Figure 2d, anti-BrdU reaction shows that in the peripheral regions of the lesion a lot of basal keratinocytes are dividing (white pentagon), thus reforming epidermal layer. BrdU staining detects nucleated cells in S-Phase which have had BrdU incorporated into their DNA. The relevant presence of these cells indicates that the epidermis is still involved in intense reproductive activity to restore the lost tissue. Similarly, under the lesion, many fibroblasts are positive to BrdU staining. The reparation process is therefore occurring and clearly not complete.

Figure 3 and Figure 4a,b illustrate the reduction of the wound area after treatment with PL and PG. The most evident difference with respect to the control samples occurred after 14 days, when both platelet derivatives gave a marked reduction of the wound area. After 18 days, the outer lesion aspect is similar for the two treatments, and suggests faster reparation process with respect to the control. This seems confirmed by histological analysis that shows a restoring process more advanced in the wounds treated with the two hemoderivatives. Some differences can however be observed between PL and PG treatments. 

Figure 3c,d shows the histological evaluation of the wounds after a 18-days treatment with PL. Figure 3c, reporting H&E staining, shows that the epidermis (bracket) appears complete and well organized in multiple layers of cells with a fair degree of keratinization. In the underlying connective tissue, there are no dermal papillae and a mild inflammatory infiltrate is visible (black arrow), as well as several blood vessels (black star). Some scattered collagen fibers are present, while the large bundles typical of the reticular layer of the dermis (white arrow) are few. There is no evidence of skin appendages such as hair follicles and glands. 

Anti-BrdU reaction shows proliferating cells (white pentagon) both in epidermal and in dermal layers of the skin (Figure 3d), indicating that some regeneration process is still occurring.

Figure 4c, referring to samples treated with PG, shows that on the surface of the sample the epidermis is completely stratified and differentiated as demonstrated by the presence of acidophilic stratum corneum. In the dermis, the presence of papillae (star) is visible and the collagen bundles are arranged as in the normal skin (not shown); inflammatory infiltrate is very modest and the formation of cutaneous annexes (hair bulbs and glands, asterisk) can be noticed. Necrotic material under reabsorption is visible. Positive to the marking with anti-BrdU are some deeper layers of the epidermis (Figure 4d). 

These results confirm the improvement of healing rate in the chosen model for both the platelet derivatives, at the tested amounts (25 µL for PL or 25 mg for PG/wound). The better performance of PG can be attributed to the continuous discharge of GFs so that it acts as a delivery system. In PL most platelets are broken during the lysis process, releasing their GFs in solution. Moreover PG, thanks to its gel-like consistency, remains at the wound site longer than PL, that less easily wets the wound after the occurrence of the eschar. These considerations can represent an explanation of the more complete restoring of the tissue integrity observed in PG histological analysis. The use of PL presents however some advantages that make it a useful alternative to PG, such as the possibility to easily subdivide it in ready to use single doses to be maintained frozen and to be quickly thawed and immediately applied during the wound medication by caregivers or by the patient itself. This approach requires however the development of formulations suitable to support PL stabilization and application, improving its contact with wound bed and optimizing therefore its efficacy.

To this aim, wound dressings loaded with PL to maintain it longer in contact with the wound site have been proposed [25]. In the present case, a bioactive dressing loaded with antimicrobial and antioxidant agents was designed to further support the PL activity. 

### 2.2. Compatibility between PL and αTphNE 

**Figure 5:**
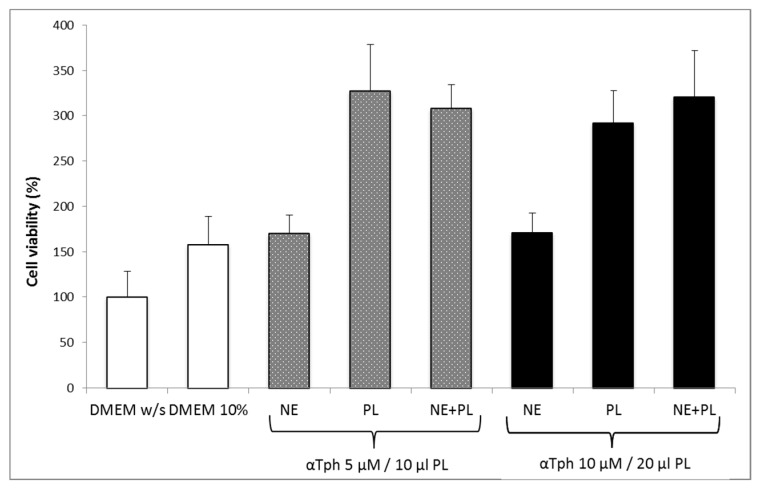
Fibroblast viability after 24 h incubation with medium without serum (DMEM *w*/*s*, control) with complete medium (DMEM 10%), with samples added to DMEM *w*/*s*: αTocopherol nanoemulsion (NE), PL, PL and αTocopherol nanoemulsion (PL + NE) (mean ± s.e., *n* = 8).

**Figure 6:**
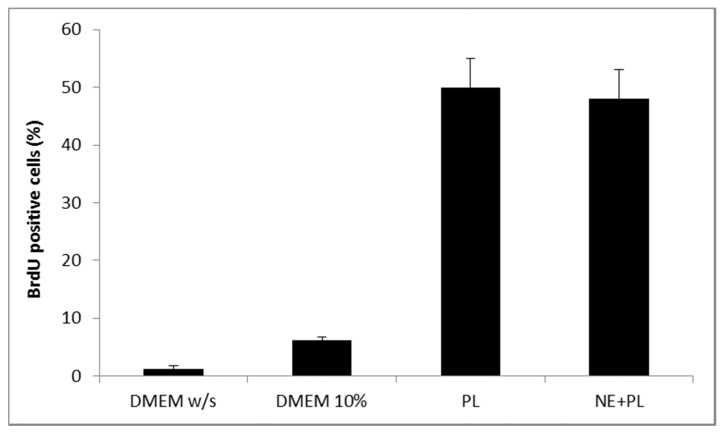
Percentage of fibroblasts BrdU positive after 24 h incubation with medium without serum (DMEM *w*/*s*, control) with complete medium (DMEM 10%), with PL and with PL and αTocopherol 10 µM nanoemulsion (PL + NE) (mean ± s.d., *n* = 3).

Figure 5 shows the results of a Neutral red viability test on fibroblasts for αTphNE at two concentrations, 5 and 10 μM, respectively, associated with 10 and 20 μL of PL to maintain a constant relationship between the concentration of αTph and the amount of PL. All the samples showed a statistically significant increase in viability with respect to the control (DMEM *w*/*s*) and no statistical difference was found between the PL and the PL + αTphNE samples at both concentrations (ANOVA one-way, post hoc Fisher test, *p* < 0.05). As already observed [27] both 5 μM and 10 μM αTph in NE showed positive effect on cell proliferation. In this case, they resulted moreover compatible with PL association. PL alone (both 10 and 20 μL) increased viability until around 300–320% with respect to the cells grown in poor medium without serum, chosen to better put in evidence the proliferation induced by the GFs present in the hemoderivative. The presence of PL allowed not only to increase viability over the negative control, but even to overwhelmingly exceed the positive DMEM 10% control, ensuring very favorable growth conditions (*p* < 0.05). These results were confirmed by evaluating the percentage of cells positive to BrdU staining after exposure to PL and to PL and αTphNE mixtures, reported in Figure 6. In addition, in this case, no statistically significant differences were observed between PL and PL mixed with αTphNE, indicating that the effect of PL on the cells is not impaired by the presence of the αTphNE. Further improvement due to αTphNE with respect to PL cannot be seen, probably due to the high stimulating effect of PL.

### 2.3. Characterization of Bioactive Dressings

**Figure 7:**
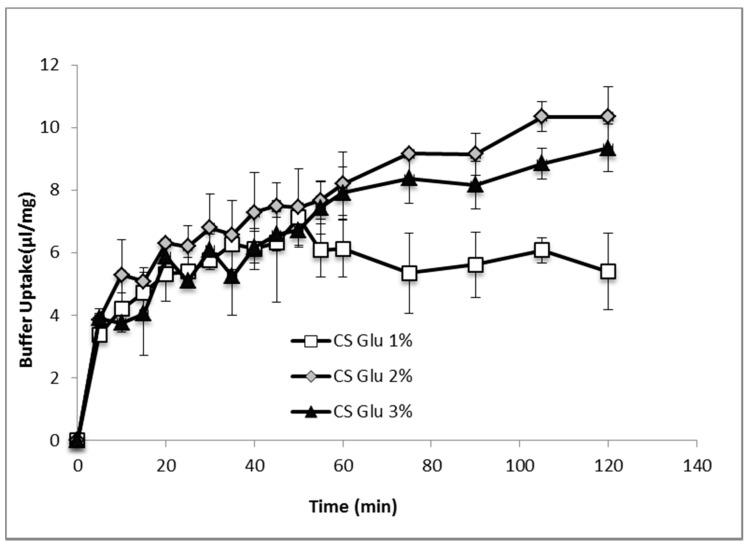
Phosphate buffer absorption profiles of bioactive dressings based on different CS-Glu concentrations (mean ± s.d.; *n* = 3).

Figure 7 shows the comparison of hydration profiles of lyophilized bioactive dressings based on 1%, 2% or 3% of CS-Glu solutions, when put in contact with phosphate buffer 0.2 M pH 7.4. CS-Glu 2% dressing showed higher absorption capacity than CS-Glu 1%, and appeared therefore interesting for its ability to load more platelet lysate and to absorb more exudate present in the wound. Further increase in absorption capacity was not observed however by increasing the percentage of chitosan glutamate from 2% to 3%, as the absorption capacity seemed even slightly lower, possibly due to more compact and less porous polymer matrix. Based on these results, the CS Glu 2% dressing was selected to continue the study.

**Figure 8:**
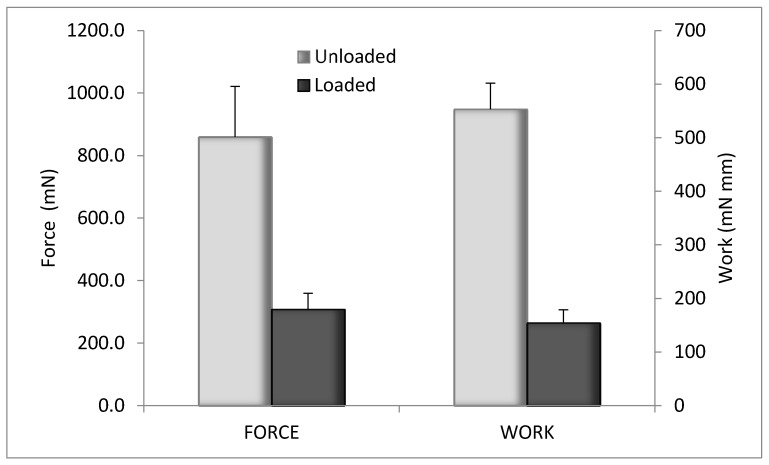
Mechanical properties of unloaded CS-Glu2% dressing and bioactive dressing, as maximum force and work of penetration (mean ± s.d.; *n* = 3).

The evaluation of the mechanical properties was performed by penetrometry test. The results are illustrated in Figure 8 for blank CS-Glu 2% and for the bioactive dressing (BD) loaded with αTphNE and AgSD-mic. Although the amount of αTph, AgSD and chitosan oleate was quite low in percentage, their presence in the dressing markedly changed the overall mechanical properties, probably for their hydrophobic cores, that interfere with the network of the CS-Glu matrix. The mechanical resistance of the BD results in fact significantly reduced compared to that of the blank CS-Glu. Despite this effect, the mechanical resistance of BD samples remained high enough to make them easy to handle and apply to the wounds.

**Figure 9:**
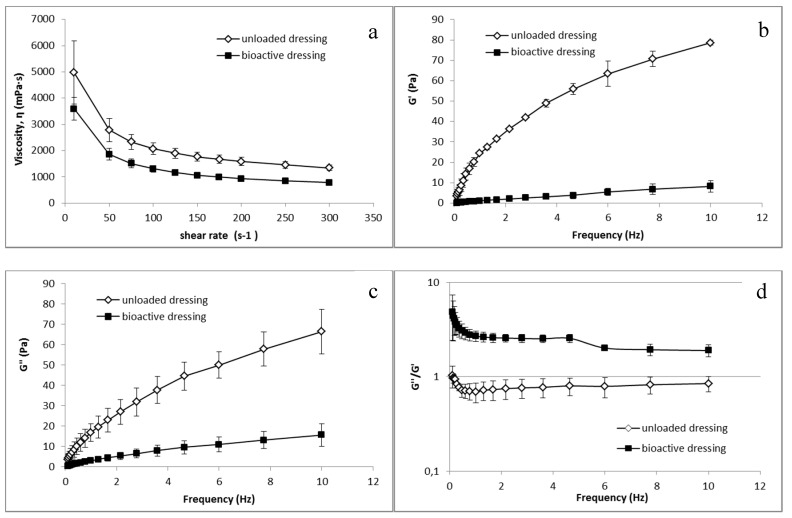
Rheological characterization of unloaded CS-Glu dressing and of bioactive dressing BD (mean ± s.d.; *n* = 3) after hydration: (**a**) viscosity curve; and (**b**–**d**) dependence of viscoelastic parameters on the oscillation frequency.

Analogous differences between CS-Glu and BD dressings were observed in the rheological tests, whose results are illustrated in Figure 9. Both unloaded and bioactive dressings, once wetted with the amount of fluid corresponding to uptake capacity, resulted in a semisolid consistency. 

Figure 9a shows the viscosity characterization, in which a clear influence of the αTphNE and AgSD-mic on the polymeric structure of the BD formulation can be observed, leading to a reduction in viscosity in comparison with the dressings made only of CS-Glu. It can be hypothesized that the presence of nanoparticles among the polymer chains reduces inter-chain interactions resulting in reduced viscosity. Figure 9b–d shows the results of the viscoelasticity characterization. G’ values describe the elastic component of the semisolid that quantify the ability of the structure to recover a deformation after relieving the stress. G” quantify the viscous, irreversible deformation. Viscoelasticity tests are more sensitive than the viscosity curves to the sample structure, and as expected the influence of the nanocarriers is even more evident in this case than in viscosity curves: the elastic modulus G’ is strongly reduced in the loaded formulation, indicating a lower elastic component. A clear reduction is visible also for the viscous component G”. However, as shown by the G”/G’ ratio, while in the CS-Glu dressing, the elastic and the viscous components are comparable, being G”/G’ ratio close to 1, the increase of this ratio in bioactive dressing demonstrates a strong prevalence of the viscous component on the elastic modulus. The presence of nanocarriers in a polymer matrix, which is already poorly structured due to the low molecular weight of the polymer and the absence of crosslinking, further reduces the elasticity of the formulation. It must be considered that low viscosity and presence of a loose structure can be advantageous for the use for which the dressing is here intended, that is to deliver nanocarriers and growth factors at the site of administration.

**Figure 10:**
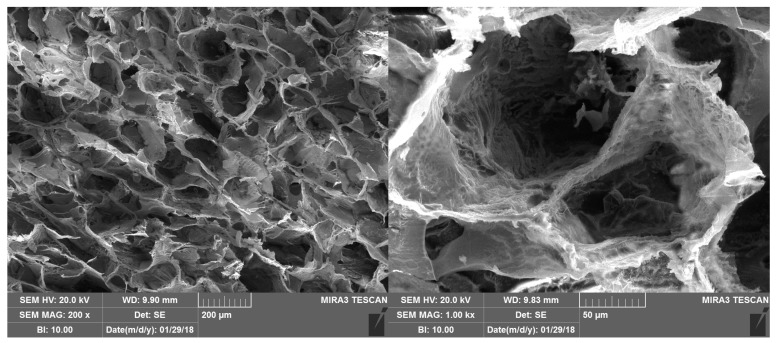
SEM microphotographs of the bioactive dressing.

Figure 10 shows the SEM morphology of the bioactive dressing, which is characterized by a regular trabecular structure with cavities of about 200 µm interconnected by small pores, quite typical of a freeze dried polymeric system. 

**Figure 11:**
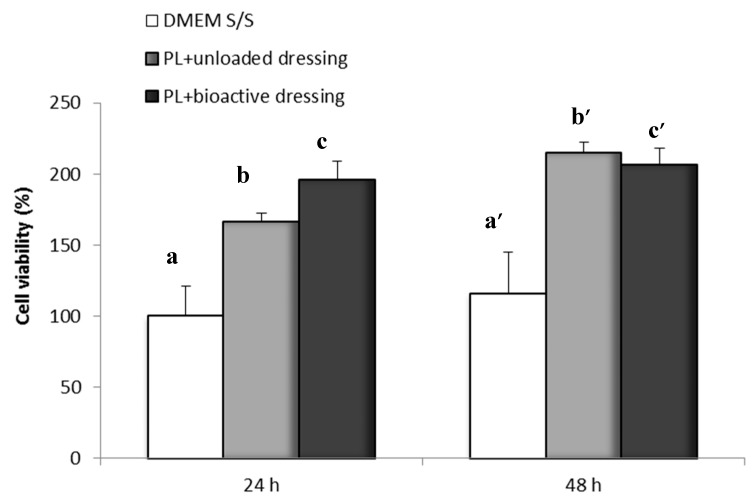
Cell viability after 24 and 48 h incubation with unloaded dressing and bioactive dressing hydrated with PL (100 µL/9 mg dressing) (mean ± s.d.; *n* = 8). Statistically significant differences (one-way ANOVA, post hoc Fisher test, *p* < 0.05) at 24 h: a vs. b, b vs. c, a vs. c; at 48 h: a’ vs. b’, a’ vs. c’.

Figure 11 shows the percentage of fibroblast viability obtained with the Neutral Red test after 24 and 48 h of incubation of the cells in the presence of PL (100 µL) loaded either on CS-Glu (unloaded dressing) or on bioactive dressings BD containing AgSD-mic and αTphNE. As described in the methods, the dressings were placed on the filters of transwell inserts, and were therefore not in contact with the cells that were seeded and grown at the bottom of the wells. After 24 h of incubation with the samples, the viability of cells exposed to the combination of PL with bioactive dressings is significantly higher (one-way ANOVA, post hoc Fisher test, *p* < 0.05) than both the control (DMEM *w*/*s*) and the PL supported by the unloaded dressing (CS-Glu). This seems to confirm the previously observed favorable effect on cell proliferation of αTphNE [27]. The results of the 48-h test confirmed the positive effect of cell growth due to the association of PL with both the unloaded and the bioactive dressing. Lack of differences can be due in this case to reaching a plateau for the strong stimulating effect of PL.

**Figure 12:**
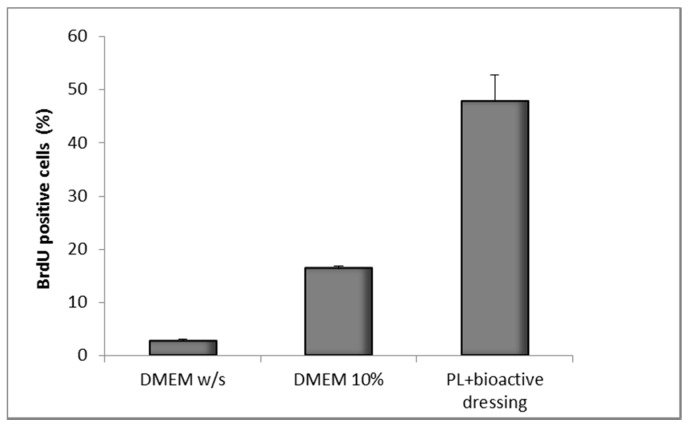
Percentage of proliferating fibroblasts (BrdU positive) after 24 h incubation with bioactive dressing hydrated with PL (100 µL/9 mg dressing) (mean ± s.d.; *n* = 3).

Figure 12 shows the results of the BrdU test on fibroblasts treated with PL supported on the bioactive dressing. The percentage of proliferating cells is 50% of the total cells, statistically higher than not only the negative control (DMEM *w*/*s*), but also the positive control with full culture medium (DMEM 10%) (one-way ANOVA, post hoc Fisher test, *p* < 0.05).

The results illustrated in Figure 11 and Figure 12 demonstrate therefore not only the compatibility of the formulation with PL, but also that the bioactive dressing is capable of releasing through the transwell membrane both PL GFs and the nanocarriers, that stimulate the proliferation of the cell substrate. This could in some way mimic the behavior of the dressing at the wound site, confirming that hydration rate and viscosity properties are suitable to bring about a good bioavailability of platelet GFs and loaded nanocarriers.

### 2.4. In Vivo Evaluation of Bioactive Dressings

**Figure 13:**
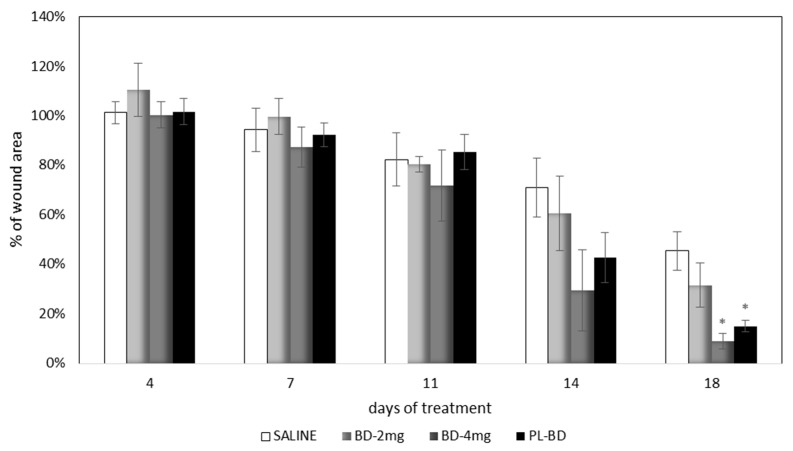
Time course of wound healing in rat model. Percentage of wound area vs. days of treatment (mean ± s.e.; *n* = 4). * indicates significant differences with respect to the control at the same time (*t* test, *p* < 0.05).

**Figure 14:**
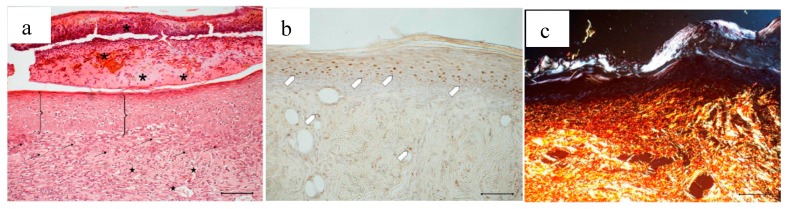
Treatment with 2 mg bioactive dressing (BD-2 mg) for 18 days. Representative images of skin slice sections stained: (**a**) with H&E; (**b**) with BrdU; and (**c**) with red picrosiurius after treatment with. Scale bar: 200 µm.

**Figure 15:**
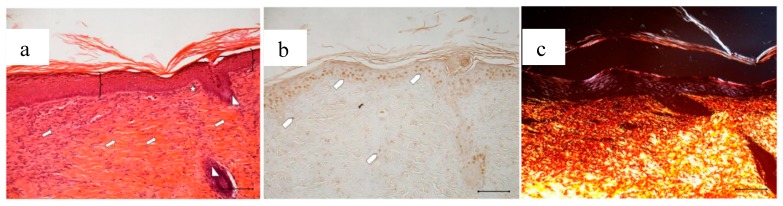
Treatment with 4 mg bioactive dressing (BD-4 mg) for 18 days. Representative images of skin slice sections stained: (**a**) with H&E; (**b**) with BrdU; and (**c**) with red picrosiurius after treatment with. Scale bar: 200 µm.

**Figure 16:**
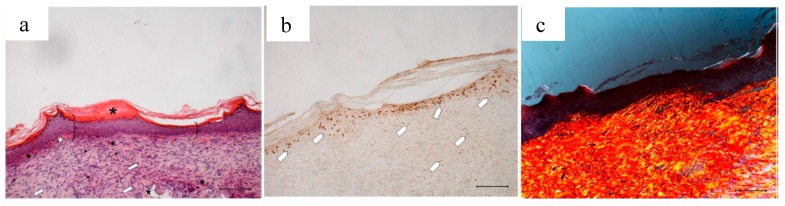
Treatment with 2 mg bioactive dressing loaded with PL (PL-BD) for 18 days. Representative images of skin slice sections stained: (**a**) with H&E; (**b**) with BrdU; and (**c**) with red picrosiurius after treatment with. Scale bar: 200 µm.

Figure 13 compares the wound area measured at different times of treatment with the control, with the bioactive dressings of different weight and with the association of BD and PL. As previously observed for the comparison between GP and PL, differences between the formulations become evident especially 14 days after treatment, although variability does not allow putting in evidence statistical differences. At 18 days, the 4 mg bioactive dressing (BD-4 mg) and the 2 mg dressing hydrated with PL (PL-BD) show the smallest wound area, in both cases significantly lower than the control (*t*-test, *p* < 0.05). For all three treatments, the histological analysis performed at 18 days, illustrated in Figure 14, Figure 15 and Figure 16, confirmed the differences observed in wound area reduction between the samples. 

In Figure 14, the histology at 18 days of a wound treated with 2 mg bioactive dressing (BD-2 mg) is given. In Figure 14a, the preparation shows a tissue reorganization that involves both the epidermis and the dermal connective tissue. The epidermal layer (bracket) is almost fully restored under a still present necrotic tissue (asterisk). There is no evidence of skin appendages as hair follicles and glands. In the dermal connective tissues, abundant granulation tissue (black arrow) and blood vessels (black star) are detectable, as well as some scattered collagen fibers that are not yet organized in the large bundles typical of the dermal layer. In anti-BrdU reaction (Figure 14b), proliferating cells appear numerous indicating restoring activity (white pentagon). Picrosirius red-staining (Figure 14c) shows the presence of many newly formed collagen fibers, which appear yellow-green colored.

Wounds treated with 4 mg bioactive dressing (BD-4 mg) after 18 days are completely re-epithelized, epidermis (bracket) is well organized in multiple layers of cells, and has a fair degree of keratinization. Even in the very middle of the scar, dermal papillae (white star) are developing, as well as dermal appendages (white triangle).

In anti-BrdU reaction, the number of proliferating cells (white pentagon) is similar to that of a mature skin. Picrosirius red-staining shows that dermal collagen appears to be rich in Green/Yellow fibers (mainly collagen III). Increasing the weight of BD applied to the wound seems therefore to improve its reparative effect, which results faster and more complete after treatment with BD-4 mg, compared to treatment with BD-2 mg.

After an 18-day treatment with PL-BD, the epidermis (bracket) appears complete and well organized in multiple layers of cells with a fair degree of keratinization. Above the epithelium, a thick layer of necrotic material (asterisk) persists. In the connective tissue, skin appendages are vacant, but dermal papillae (white star) begin to reform, while they were still absent in wounds treated with just PL (Figure 4a). With respect to PL alone, some differences can be found in the organization of collagen fibers that appear here better organized in large bundles (white arrow) typical of the reticular layer of the dermis. In anti-BrdU reaction, some keratinocytes as well as some fibroblasts are immunodetected (white pentagon). In line with that observed in Figure 16a, picrosirius red-staining (Figure 16c) highlights the mature collagen bundles in red. These results confirm an improvement of the healing efficacy for PL when supported on BD-2 mg. This can be ascribed to different mechanisms, such as a protection of GFs by chitosan, the improved contact of PL supported on dressing with the wound, or the synergic effect of PL GFs with the antioxidant and with chitosan and chitosan oleate components of the bioactive dressings. These findings suggest therefore the usefulness of further future studies about the specific role that each of these components play in wound healing process.

## 3. Materials and Methods 

### 3.1. Materials

The following materials were used for the encapsulation of AgSD and αTph: Chitosan (CS) was obtained as HCl salt from low molecular weight (LMW, 50–190 KDa) chitosan base, deacetylation degree 80% (Sigma Aldrich, Milan, Italy) by addition of HCl 0.5 N to chitosan until complete dissolution, dialysis in bidistilled water for 24 h and freeze-drying (Heto Drywinner, Analitica de Mori, Milan, Italy). Oleic acid was from Fluka (Milan, Italy), α-tocopherol (αTph) and AgSD were from Sigma-Aldrich (Milan, Italy). For the dressings, low molecular weight chitosan (about 250 kDa) deacetylation degree 98% (Primex chitoclear TM 1504, Giusto Faravelli, Milan, Italy) was used. Glutamic acid and glycine were from Sigma-Aldrich (Milan, Italy). Materials used for cell culture tests and in vivo studies were obtained from Sigma Aldrich (Milan, Italy), unless otherwise specified.

### 3.2. Platelet Lysate and Platelet Gel Preparation

Platelet lysate (PL) and platelet gel (PG) were supplied by Immunohematology and Transfusion Service and Cell Therapy Unit of Fondazione IRCCS, S. Matteo, Pavia, Italy. A pooled sample prepared from platelet rich plasma (containing 700 × 10^3^ platelets/µL) obtained from eight different blood donors was used to prepare both the derivatives. To obtain platelet lysate (PL), aliquots of platelet pool were subjected to two cycles of freezing at −80 °C for 5 h and thawing in a sterile water bath at 37 °C. To obtain the platelet gel (PG), 1 part thrombin and 0.5 parts calcium gluconate were added to 3 parts platelet lysate, according to [36]. 

### 3.3. Dressing Preparation and Characterization 

#### 3.3.1. Dressing Composition

Chitosan glutamate salt (CS-Glu) was prepared by adding glutamic acid to a chitosan dispersion 1% *w*/*v* in water. The excess of glutamic acid was removed by dialysis and the final solution was freeze dried (Heto Drywinner, Analitica de Mori, Milano, Italy). 

The preparation and characterization of the nanocarriers was previously described [26,27], and it is here briefly repeated. Acetone solutions of either AgSD (2 mg/mL) or alpha tocopherol (αTph) (1 mg/mL) and oleic acid (10 mg/mL) were prepared and dropwise added to 0.5 mg/mL aqueous solutions of chitosan HCl under stirring to obtain chitosan (CS):oleic acid (OA) ratio 1:1 *w*/*w*. Acetone was removed by evaporation. The samples were sonicated 15 min (Elmasonic S 80 H, Elma Hans Schmidbauer GmbH & Co, Singen, Germany) before characterization. Particle size characterization was performed by photon correlation spectroscopy (PCS) (N5 Submicron Particle Size Analyser, Beckman Coulter, Milan, Italy), and it was verified that the dimensions were in line with the expected values (between 150 and 250 nm). 

Dressings were prepared by redissolving chitosan glutamate 1%, 2% and 3% *w*/*v* and adding glycine as cryoprotectant at 1% of the final dressing weight. To obtain loaded bioactive dressings, AgSD-Mic [26] and αTphNE [27] were added to final AgSD and αTph amounts in accordance with Table 1. Final dispersions were freeze dried in vials to obtain dressings of about 0.95 cm^2^ area.

#### 3.3.2. Hydration Test

Each dressing of composition according to Table 1 was put on a filter paper and maintained in contact with phosphate buffer 0.2 M pH 7.4. At defined times, the dressing was weighed to assess the amount of water absorbed. Three replicates were performed for each formulation.

#### 3.3.3. Morphology

Dressing morphology was analyzed by means of scanning electron microscopy (SEM, Tescan, Mira3XMU, ARVEDI Center, University of Pavia, Pavia, Italy). Samples were sputtered by means of graphite deposition under vacuum.

#### 3.3.4. Characterization of Mechanical Properties

A breaking force test was performed by a Texture Analyser Stable Micro Systems TA.XT Plus (ENCO, Spinea, Venezia, Italy), using a penetration test setting: cylindrical probe (5 mm diameter), trigger force of 0.1 g, loading cell of 1 kg, 1 mm/s rate, at a distance of 1.5 mm. The probe was lowered towards the dressing supported on a ring. As response the breaking force was measured (mN), and the work was calculated as area under the force-displacement curve (mN·mm). 

#### 3.3.5. Rheological Characterization

Dressings were hydrated with 100 µL of phosphate buffer 0.2M, pH 7.4, and subjected to rheological characterization by means of a rotational rheometer (Rheostress 600, Haake, Karlsruhe, Germany) equipped with a cone/plate measuring system C1°/35 mm (angle = 1°, diameter = 35 mm), at 32 °C. Viscosity was measured in the 10–300 s^−1^ shear rate range. 

Viscoelasticity tests were performed within the linearity range previously detected. Oscillation test was performed at 0.7 Pa between 0.1 and 10 Hz frequency. Elastic modulus G’, viscous modulus G” and the G”/G’ ratio (tgδ) were measured. 

### 3.4. In Vitro Evaluation on Cell Cultures

Normal Human Dermal Fibrobasts (NHDF, Promocell) were grown in Dulbecco’s Modified Eagle’s Medium (Lonza, Milan, Italy) supplemented with 10% (*v*/*v*) Fetal Bovine Serum (Euroclone, Milan, Italy), 1% *v*/*v* Penicillin–Streptomycin 100× and 1% (*v*/*v*) Amphotericin (WVR, Milan, Italy). Fibroblasts were maintained in a humidified atmosphere with 5% CO_2_ at 37 °C and routinely split every 7 days using Trypsin-EDTA solution 0.25%. The cells used were between the 5th and the 13th passage.

#### 3.4.1. Proliferation Test 

The proliferation test for the αTphNE and PL (biocompatibility test) was performed on fibroblasts in 96 wells plates CELLSTAR, Greiner Bio-one GmbH, Frickenhausen, Germany). Twenty thousand cells/well were seeded, added with 200 µL of the samples diluted in culture medium without serum (DMEM *w*/*s*) and incubated 24 h at 37 °C. Cell viability was assessed by means of both Neutral Red test and BrdU assay.

**Figure 17:**
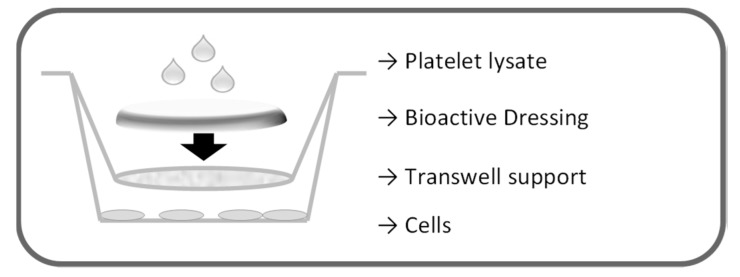
Schematic representation of the setting of the proliferation test for the dressings.

The proliferation test for the dressings was performed on fibroblasts in transwell 0.40 µm pore size, 1.12 cm^2^ insert area (Constar, Corning, NY, USA), as illustrated in Figure 17. First, 2.26 × 10^5^ cells/well were seeded in 12 well plates with 1.5 mL culture medium without serum (DMEM *w*/*s*). Dressings (0.95 cm^2^) were placed on the transwell membranes that were introduced in the wells to avoid contact of the formulation with the cell layers. Each dressing was therefore wetted with 100 µL either of PBS or of platelet lysate. Cells were incubated 24 h, and cell viability was assessed by means of both Neutral Red test and BrdU assay.

For the Neutral Red test, the medium was removed, fibroblasts were washed with PBS and treated with 200 µL of Neutral Red 0.5 mg/mL in HBSS. After 3 h, the medium was removed, and the cells were washed with PBS. Two hundred microliters of 50% *v*/*v* ethanol and 1% *v*/*v* glacial acetic acid were added to each well to disrupt cell membranes and solubilize NR present in vital cells. The plate was kept away from light for 5–10 min and the absorbance was read by a Microplate Absorbance Reader iMARK™ (Bio-Rad Laboratories S.r.l., Segrate, MI, Italy) at a wavelength of 490 nm. The percentage of viability was calculated considering as 100% the viability of the control represented by the culture medium without serum (DMEM *w*/*s*). 

#### 3.4.2. Bromodeoxyuridine Test

For Bromodeoxyuridine (BrdU) test, cells were seeded in growth medium without serum (DMEM *w*/*s*) on 12 mm diameter round slides introduced at the bottom of each well. One hour before the end of the test, 20 μL of BrdU were added at 30 µM final concentration. Cells were washed with PBS, fixed with 70% ethanol and stored at −20 °C. The incorporated BrdU was detected by an immunostaining reaction with Amersham monoclonal anti-BrdU antibody (GE Healthcare UK Ltd., Amersham Place, Buckinghamshire, UK). Briefly, the dishes were washed with PBS and incubated with HCl 2 N for 30 min at room temperature. Sodium tetraborate (Na_2_B_4_O_7_·10H_2_O, pH 8.5, 0.1 M) was used to neutralize the solution for 15 min, and then the cells were washed twice for 5 min in PBS and incubated for 20 min in the blocking solution (1% *w*/*v* BSA and 0.02% *w*/*v* Tween 20 in PBS Tween Albumin (PTA)). Cells were then incubated for 1 h with mouse anti—BrdU antibody diluted 1:100 in PTA. The cells were washed three times (10 min each) in PTA and then incubated again for 30 min in PTA containing anti-mouse IgG FITC-antibody (Sigma-Aldrich, Milan, Italy) diluted 1:100. The slides were extensively washed in PBS, counterstained for DNA with 0.5 μg/mL Hoechst 33258 (Sigma-Aldrich, Milan, Italy), and mounted in Mowiol (Sigma-Aldrich, Milan, Italy). Detection was performed with a fluorescence microscope Zeiss Axiophot (Carl Zeiss, Oberkochen, Germany) by evaluating the ratio of the number of proliferating cells (green, as positive to BrdU) on the total number of vital cells (blue nuclei, stained with Hoechst).

### 3.5. In Vivo Studies

#### 3.5.1. Evaluation on Rat Wound Model

All animal experiments were carried out as previously described [35] in full compliance with the standard international ethical guidelines (European Communities Council Directive 86/609/EEC) approved by Italian Health Ministry (D.lgs.vo 116/92). The study protocol was approved by the Italian Health Ministry (77/2013-B, 25 March 2013). 

In brief, male rats (Wistar 200–250 g) were anesthetized with equitensine (3 mL/kg) and shaved to remove all hair from the site of injury. Three 4 mm-full thickness burns were produced on animal back by contact with a brass rod (105 °C for 40 s). Twenty-four hours later, three 6-mm full-thickness excisional wounds were outlined using a punch biopsy tool on each animal back. In a preliminary part of the study, involving 3 rats, wounds were treated either with 25 µL of PL or with 25 mg PG. Twenty-five microliters of saline solution (NaCl 0.9% *w*/*v*) were used as treatment of controls. Each rat was treated with saline, PL and PG (*N* = 3 for each sample). In the second part of the study, involving other 6 rats, the developed bioactive dressings were tested, and comparison was performed between bioactive dressings in two different weights, 2 and 4 mg (BD-2 mg; BD-4 mg), PL-loaded bioactive dressing (PL-BD, that is BD-2 mg wetted with 25 µL of PL), or saline solution (25 µL) as control. The dressings were placed on the wound and hydrated with either saline (BDs) or PL (PL-BD). All the rats were treated with saline (control), while the other two burns received different treatments (*N* = 4 for each treatment). Each wound was covered with a sterile gauze and the rat’s back was wrapped with a surgery stretch (Safety, Monza, Italy) to protect lesions. At prefixed times (0, 3, 7, 10, 14 and 18 days) each lesion was photographed with a digital camera (Sigma SD 14) to monitor the healing process and treated either with one of the formulations or with saline. The photographs were analyzed using UTHSCSA Image Tool v. 3.0 software (The University of Texas Health Science Center, San Antonio, TX, USA). A wound healing >80% was considered the endpoint. Eighteen days after the treatment, full thickness biopsies were obtained and a histological analysis of the excised tissues was carried out.

#### 3.5.2. Histological Analysis

After 18-day treatment, the animals were in vivo labeled with BrdU by intraperitoneal injection of a sterile solution of 10 mg/mL BrdU (5-Bromo-2′-deoxyuridine, Sigma, Milan, Italy), 100 mg/kg. After 60 min the animals were sacrificed and tissue samples were bisected along the widest line of the wound, then fixed in 4% *w*/*v* neutral buffered paraformaldehyde for 48 h, dehydrated with gradient alcohol series, cleared in xylene and embedded in paraffin. Sections (8 μm) were obtained using a Leitz (Wetzlar, Germany) microtome and were stained with hematoxylin and eosin (H&E) or subjected to imunohistochemical detection of BrdU. 

For BrdU staining, sections were deparaffinized in xylene and hydrated in a series of graded alcohols to water, then slides were immersed in a blocking reagent (Biocare’s Peroxidazed 1 blocking reagent, Biocare Medical, Pacheco, CA, USA) for 5 min at RT and subsequently washed in distilled water. For heat-mediated antigen retrieval slides were placed in Rodent Decloaker buffer (Biocare Medical, Pacheco, CA, USA) and heated to 100 °C for 30 min by a steamer; when slides became cold Rodent Block M (Biocare Medical, Pacheco, CA, USA) was applied for 30 min. After Tris-buffered saline (TBS) washing, primary antibody (monoclonal anti Bromodeoxyuridine, Biocare Medical, Pacheco, CA, USA) was applied 1:100 for 2 h at room temperature. Samples were subsequently washed in TBS and then covered with Mouse-on-Mouse HRP-Polimer (Biocare Medical, Pacheco, CA, USA) for 20 min at room temperature. After a thorough washing in TBS, slides were incubated for 5 min with chromogen solution (Biocare’s DAB, Biocare Medical, Pacheco, CA, USA). Positive and negative controls were performed as well.

For collagen detection in tissue sections, some slides of the wounds treated with the dressings were stained with picrosirius red (Sigma Aldrich Direct Red 80) for one hour, and then washed in acidified water (5 mL acid acetic to 1 L of water). The slices were examined at the magnification of 5× under a light microscope Axiophot Zeiss (Oberkochen, Germany) equipped with crossed polars and digital camera.

## 4. Conclusions

The results show that the encapsulation of poorly soluble hydrophobic molecules in chitosan oleate allows easy dispersion in hydrophilic formulations such as freeze-dried dressings. In the present case, the association of chitosan oleate nanocarriers containing AgSD and αTph resulted in a dressing whose effect on wound healing was dose dependent, as faster recovery of the wounds was observed after application of 4 mg (BD-4) compared to 2 mg (BD-2) dressing. This seems to confirm the bioactive behavior of the formulation, due to combined activity of chitosan and chitosan oleate associated to the presence of the antioxidant. The positive in vivo results confirmed the protective effect of encapsulation towards AgSD denaturing effect, and the positive stimulation of αTphNE towards keratinocytes and fibroblasts, previously observed in vitro. The combined application of PL with bioactive 2 mg dressing (PL-BD) significantly improves the dressing effect and an improvement of PL efficacy on wound healing promotion is also suggested. The bioactive dressing can therefore be a suitable and versatile support of the hemoderivative for the treatment of wounds. The dose dependent effect of the bioactive dressing makes it a flexible tool in wound treatment. The combination of PL with the bioactive support can in fact be modulated to obtain the best healing effect with the lowest PL amounts. Moreover, as PL preparation involves freezing steps, hemoderivative doses can be safely stored, handled and associated to the dressings without efforts by caregivers or patients. 

## Figures and Tables

**Table 1:** Composition of the bioactive dressings.

Components	CS-Glu 1%	CS-Glu 2%	CS-Glu 3%
CS glutamate (mg)	3.79	8.74	13.69
CS oleate (mg)	0.09	0.09	0.09
Alpha tocopherol (mg)	0.07	0.07	0.07
Ag sulfadiazine (mg)	0.01	0.01	0.01
Glycine (mg)	0.04	0.09	0.14
Total weight	4.00	9.00	14.00
